# State-of-the-art Laser Ablation for Cardiac Arrhythmias: Development and Future Perspective—A State-of-the-art Review

**DOI:** 10.19102/icrm.2025.16124

**Published:** 2025-12-15

**Authors:** Helmut Weber, Lutz Ruprecht, Michaela Sagerer-Gerhardt

**Affiliations:** 1 Research Development Department, CCEP Center Taufkirchen, Taufkirchen, Germany; 2 Central Laser Laboratory, Helmholtz Center Munich, Munich, Germany; 3 Laboratory Animal Facilities, Helmholtz Center Munich, Munich, Germany

**Keywords:** 1064-nm laser, atrial fibrillation, cardiac arrhythmias, ganglion plexi, laser catheter ablation

## Abstract

Cardiovascular laser application (CVLA) is an innovative approach in the field of cardiology aimed at treating various cardiovascular diseases, including cardiac arrhythmias, cardiomyopathies, systemic and pulmonary resistant hypertension, and varicose leg veins, by using a key technology, the laser. Tissue-selective photon absorption of the 1064-nm wavelength induces tissue-selective irreversible lesions of arrhythmogenic myocardium, modulation of retrocardiac ganglion plexi, and renal and pulmonary perivascular innervation. This review summarizes the development and the experimental and clinical results of the CVLA, highlighting its potential advantages over other catheter ablation methods. Based on its unique characteristics, laser treatment has the potential to become an all-pervasive, safe, and effective procedure for the benefit of countless patients.

## Introduction

For catheter ablation of cardiac arrhythmias, a variety of catheter ablation modalities are used.^1,2^ However, treatment success remains limited even after innovative approaches, with pulsed field ablation yielding a 1-year arrhythmia-free outcome of only 53% per Kaplan–Meier analysis^3^ and associations with complications such as transient and—in rare cases—persistent phrenic nerve palsy, transient ischemic attacks, stroke, severe coronary arterial spasm resulting in ST-segment elevation, and atrioventricular (AV) block despite remote distance from the ablation site. In addition, hemolysis-related acute renal failure necessitating hemodialysis, pericardial tamponade, microembolic signals frequently clustered in short-lasting shower-like patterns with unknown impacts on neurological outcomes and cognitive decline, and severe neurovascular events and death can occur.^4–8^ To cope with these limitations, a novel cardiovascular laser application (CVLA) system—the non-contact, open-irrigated, high-density (HD) laser-mapping-guided RytmoLas^®^ catheter (LasCor GmbH, Taufkirchen, Germany)—has been developed.^9^

## Parameters of cardiovascular laser application

### Lesions

#### Lesion formation

Owing to a low absorption coefficient of the 1064-nm laser photons in water,^10^ photons are not absorbed by transparent/translucent tissue such as the endo- and epicardium but are selectively absorbed by turbid tissue such as the myocardium. Tissue-selective absorption of the 1064-nm laser in myocardium is one of the unique advantages of CVLA.

Laser ablation is performed under normothermic conditions. The catheter itself is not heated up; it does not transmit heat. Lesions are induced deep intramurally and grow gradually, resulting in a homogenous solid volume of coagulation necrosis with well-demarcated boundaries.^11,12^ Irreversible thermal effects on the myocardium begin at temperatures of 50°C. Excessive tissue temperatures of ≥100°C should be avoided because of thermal damage caused by tissue overheating.

**Figure 1:**
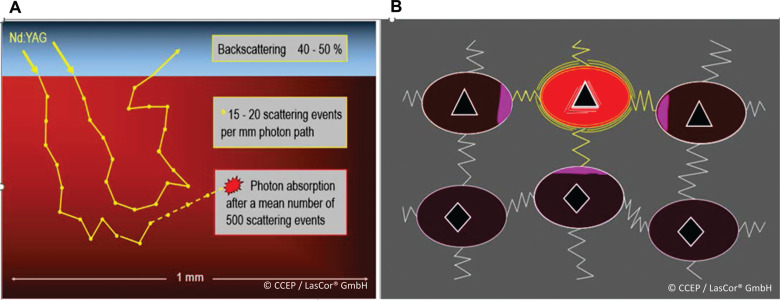
Photon scattering of the 1064-nm laser light. A schematic representation is shown. **A:** Scattering and selective absorption of the 1064-nm laser photons in myocardial tissue. **B:** Only photons of appropriate wavelength are absorbed by the corresponding molecules. Their energy is transformed into molecular oscillation, inducing heat by friction between molecules.

**Figure 2:**
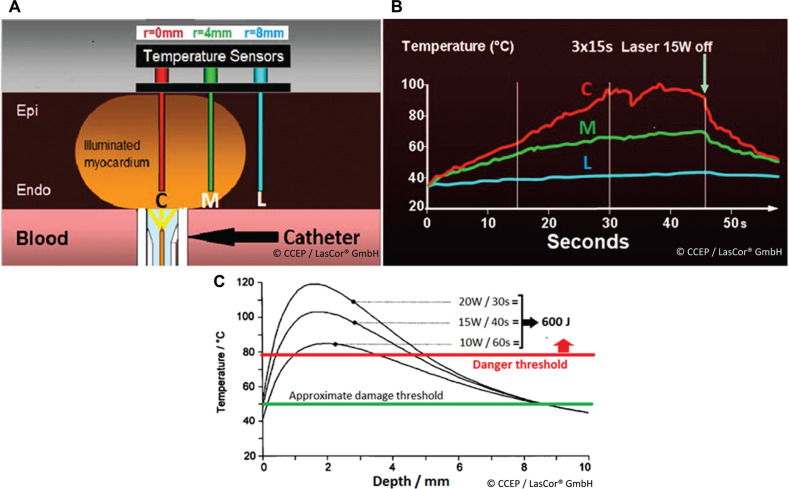
Intramural myocardial temperature measurement during laser. A schematic representation is shown. **A:** Sensor array. **B:** Temperature curves during the 1064-nm laser application aimed at the endocardial surface of the left ventricular free wall in a dog model showing a continuous rise of temperature with the highest value along the beam axis *r* = 0 mm. C represents central, M is medial, and L is the lateral sensor with 4.0 mm in between. **C:** Temperature induced at 600 J with various energy settings. The danger threshold is considered to be at 80°C.

Photons of the 1064-nm laser light are mainly scattered; roughly 40%–50% are backscattered or are absorbed after a mean number of 500 scattering events **(Figure 1A)**. Only photons of appropriate 1064-nm wavelengths are absorbed by the corresponding molecules, inducing heat by friction between the molecules **(Figure 1B)**. For the measurement of myocardial heat induction, a sensor array inserted epicardially in the left ventricular free wall of an anesthetized dog heart has been used **(Figure 2A)**. During three consecutive 15-W/15-s laser applications, a gradual temperature increase of the myocardium with a maximum of 90°C after 35–40 s has been registered **(Figure 2B)**. With an unchanged 600-J application, temperature curves differ substantially with various energy settings **(Figure 2C)**. This experiment allowed for narrowing down the ranges of laser power and radiation time. Within these limits, sufficiently high temperatures for myocardial coagulation can be achieved for transmural lesions within seconds without the risk of collateral damage.^13^

Intramural heat generation has been simulated in a Monte Carlo model as a function of the laser beam profile and divergence versus individually varying tissue properties. A catheter concept for safe endocardial photocoagulation (RytmoLas^®^) has been introduced, encompassing a highly divergent beam of maximized spot size, effective catheter flushing, and shielding of blood (due to mounting of the optical fiber coaxially in the central lumen of the catheter tube with its tip at 1.0–1.2 mm from the endhole of the open irrigated catheter). Lesion formation is not influenced by beam parameters but is mainly determined by the optical tissue properties—the tissue-selective absorption of photons.^14^

After laser application, the acute lesion still grows slightly due to heat conduction. It can take several days to heal to a dense fibrous scar, so electrophysiological follow-up examination in the same session can lead to false results and can unnecessarily stress and endanger the patient. Instead, the result of the treatment only becomes known after weeks or months. Appropriate monitoring measures during the monitoring period include clinical checks, blood pressure measurements, long-term and event electrocardiogram and cardiac monitors, telemetry, and exercise tests. Invasive electrophysiology is justified and can be performed in symptomatic patients, with laser treatment repeated if necessary.

#### Lesion quality

**Figure 3:**
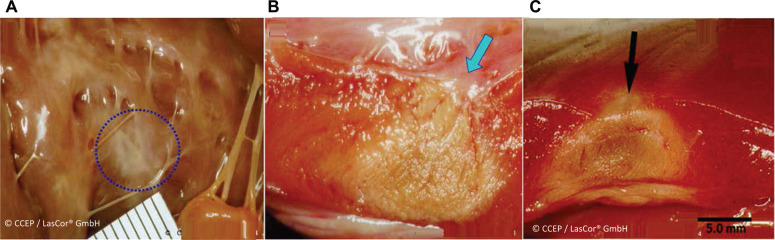
In vivo transcatheter ventricular 1064-nm laser lesions at 15 W/20–30 s in a dog model. **A**, **B:** Clear-cut, homogenous, transmural lesions of coagulation necrosis, produced by an endocardial approach. **C:** Epicardial approach, involving 1064-nm laser application at 15 W/30 s in the left ventricular free wall in a dog model. The arrows indicate catheter orientation during laser application. Note the endo- and epicardial layers without tissue vaporization and crater formation.

**Figure 4:**
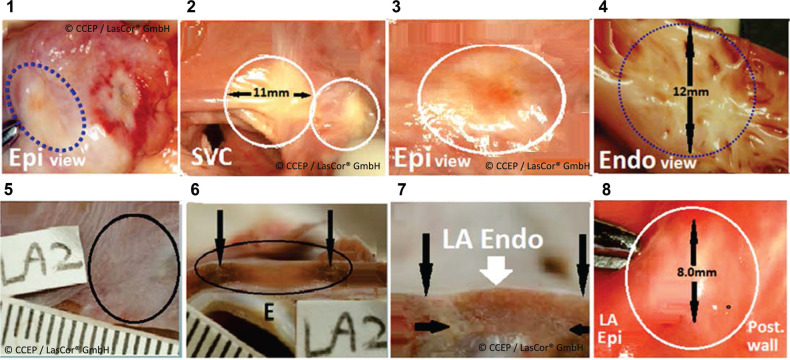
In vivo transcatheter atrial laser lesions at 15 W/6-10 s in a dog model. **(1)** Transmural scar in the right atrial (RA) free wall (dotted circle) and an acute lesion (hemorrhagic margins) in the basis of the RA appendage. **(2)** Endocardial scar in the RA roof (11.0 mm) and septum (white circles). **(3)** Transmural scar in the RA roof (white circle). **(4)** Scar in the trabeculated RA area (12.0 mm). Transmural scars in the left atrial posterior walls. **(5)** Endocardial view (black circle), cross-section, **(6)** black oval, **(7)** white arrow, and **(8)** transmural lesion (white circle). *Abbreviations:* E, esophagus; Epi, epicardial; Endo, endocardial; LA, left atrium; SVC, superior vena cava.

**Figure 5:**
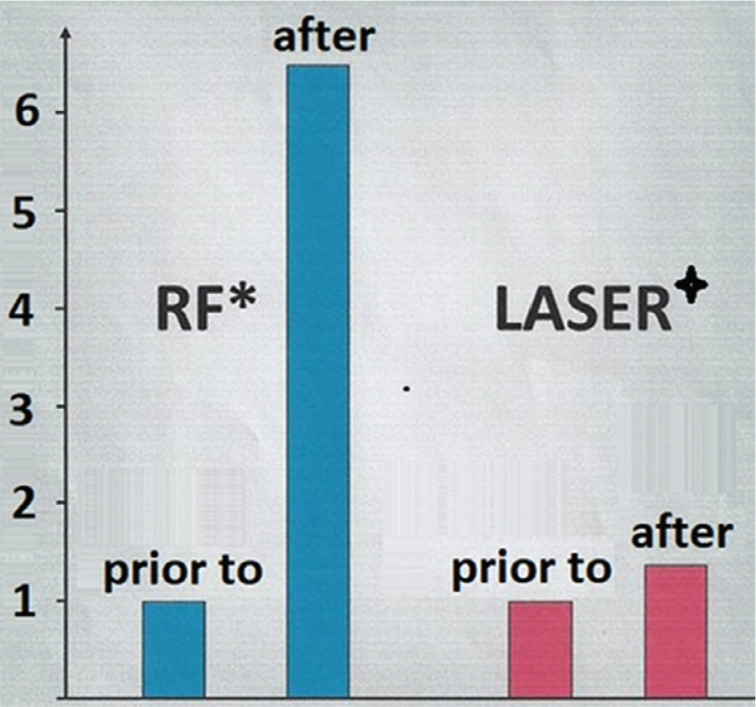
Comparison of serum D-dimer levels between radiofrequency and laser ablation, before and after the procedure. *Radiofrequency data sourced from Manolis AS, Melita-Manolis H, Vassilikos V, et al. Thrombogenicity of radiofrequency lesions: results with serial D-dimer determinations. *J Am Coll Cardiol*. 1996;28(5):1257–1261. ^+^Laser data sourced from Zhuang S, Weber H, Heinze A, Wanner G, Weis L. D-Dimer serum level after laser catheter ablation of tachyarrhythmias. *Pacing Clin Electrophysiol*. 1999;22:A94/P196.

Due to selective absorption and homogenous distribution of the 1064-nm laser photons in the myocardium, the result is a clear-cut, homogenous lesion of coagulation necrosis without tissue vaporization with crater formation. With the abolishment of electrical potential amplitudes in the HD laser-mapping, lesions are transmural **(Figure 3)**. The same is also the case after laser applications aimed at the atrial walls. Generally, extended, contiguous, overlapping transmural atrial lesions can be achieved within seconds. Acute lesions will heal without shrinking or the formation of an aneurysm **(Figure 4)**. As a result, when properly applied and executed, the laser renders a negligible risk of sustaining re-entry pathways or arrhythmogenic foci within the treatment area. Laser lesions are not arrhythmogenic and, as reflected by unchanged D-dimer serum levels, are not thrombogenic **(Figure 5)**, either immediately or long term—a unique characteristic of the laser method.^15,16^ For effective treatment, extensive shallow and transmural lesions are desired, limited to the culprit arrhythmogenic substrate in the myocardial wall while saving the surrounding healthy myocardium and adjacent mediastinal structures such as the esophagus, lungs, and nerves.

#### Lesion size and shape

**Figure 6:**
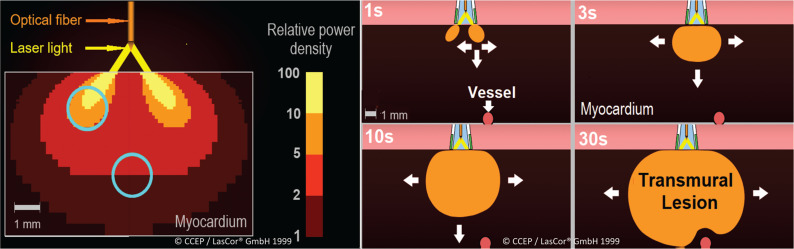
Lesion growth during laser application. Schematic Illustration of transcatheter laser lesion formation by selective photon absorption in myocardium. In deeper regions, light transport is mainly diffuse. Lesions are still growing 1–2 mm by heat conduction 5–10 min after radiation.

Critical to the success of laser ablation is the size and shape of the lesions produced. Regardless of thin-walled arrhythmogenic substrates located in the atrial wall, or those in the thick-walled ventricular myocardium, for safety reasons, transmural lesions limited to the myocardial wall are desirable. In general, with circular, donut-like radiation of the endocardial surface, lesion formation starts deep intramurally and extends gradually, resulting in a bowl- to spherical-shaped dense coagulation volume **(Figure 6)**.

However, configuration of arrhythmogenic substrates my vary widely, extending deep into myocardial walls and misrouting excitation propagation, which may require deep and extensive transmural lesions for successful ablation. In general, for long-term success of treatment, transmural lesions are needed. Significantly larger lesions can be achieved with the CVLA system compared to radiofrequency (RF) ablation.^17^

### Catheter parameters

#### Catheter irrigation

When using an open-ended catheter in the bloodstream, continuous catheter irrigation is a must. With insufficient irrigation, penetration of blood into the catheter will instantly burn down the fiber tip, which otherwise is not heated up during laser applications. Heat is produced deep intramurally by selective absorption of 1064-nm laser photons by myocites. Penetration of blood into the catheter will cause blood clot formation, burning, and carbonization of the distal catheter end and will destroy the catheter and endanger patients. Higher irrigation flow more effectively washes away the blood, creating a transparent path for the laser light with a better photon transmission to the targeted myocardial wall, thus producing larger lesions. However, a back-thrust flow of the saline jet from the end-hole of the catheter that is too vigorous may destabilize the catheter and potentially endanger patients by volume overload.

**Figure 7:**
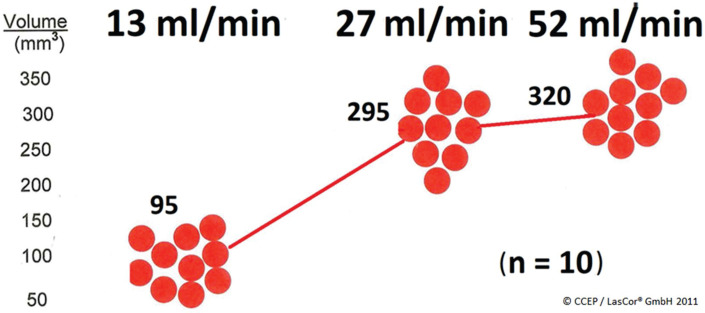
Influence of catheter irrigation on lesion formation. Mean values of lesion volumes in mm^3^ produced at 15 W/15 s of continuous-wave 1064-nm laser application in bovine myocardium during various catheter saline flow rates by using the RytmoLas^®^ catheter.

Open-irrigated laser catheter ablation produces flow-dependent sizes of lesions.^18^ The optimal flow rate of RytmoLas^®^ produces lesions in a non-contact mode of radiation.^19^ Catheter flow between 30 and 50 mL/min does not substantially increase lesion size **(Figure 7)**. To avoid patient volume overload during the laser treatments, optimal continuous catheter flow was found to be 10 mL/min, and high flow during laser application was 35 mL/min. These values are preset in the laser software for control of saline irrigation via the laser foot switch. The automatic increase of flow via the laser foot switch creates an optimal, clear pathway for the laser light to the targeted endocardial area. In addition, catheter irrigation cools the irradiated surface. The decisive factor for the cooling effect is not the irrigation rate but rather the room temperature (18°C) of the irrigation fluid. In the context of the usual fluctuations in room temperature, the influence of the irrigation temperature is insignificant.

#### Contact force (CF)

**Figure 8:**
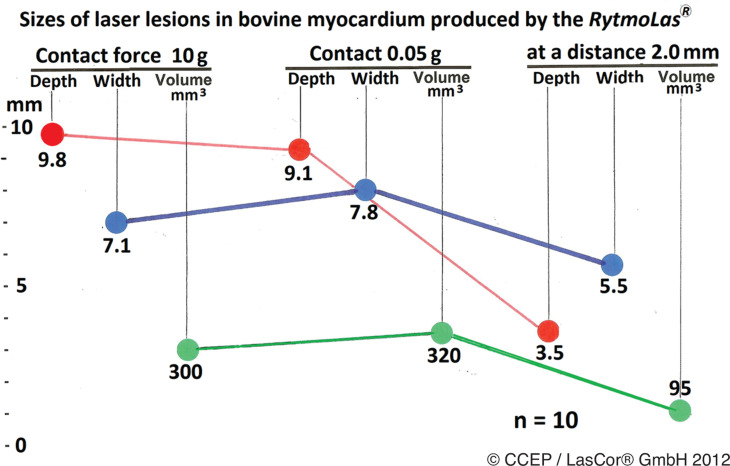
Influence of catheter contact force on laser lesion formation. Mean values of lesion sizes produced at 10 W/30 s in blood at 10°C; catheter irrigation, 30 mL/min.

During RF and all the other catheter ablation methods, CF on the myocardial wall is needed for a substantial increase in lesion size. However, CF may result in severe myocardial injury, including wall perforation. By using RytmoLas^®^, catheter–tissue CF is not a major determinant for lesion size and quality. Maximum sizes of lesions can be achieved with the catheter in intimate contact with the endocardial target area in the beating heart. There were no significant differences between volumes of lesions achieved with a catheter–tissue CF of 100 g (297 ± 56.0), 10 g (300 ± 39), and in contact without pressure (320 ± 24), respectively (*P* > .05). However, volumes of lesions produced at a catheter distance of 2 mm (95 ± 14 mm^3^) were significantly smaller (*P* < .0001) **(Figure 8)**, whereas no lesions were produced at 5.0 mm. No mural thrombi or a steam pop with a crater or thrombus formation occurred. Non-contact laser radiation through a clear saline irrigation corridor with a divergent laser beam allows for a “donut”-like light spot with a homogenous power density without gaps in the irradiation field. No thrombus formation occurred during laser application with the free-floating laser catheter in stagnant blood.^20,21^ However, a stable orientation of the catheter during laser application is needed for adequate lesion formation.

#### Orientation

**Figure 9:**
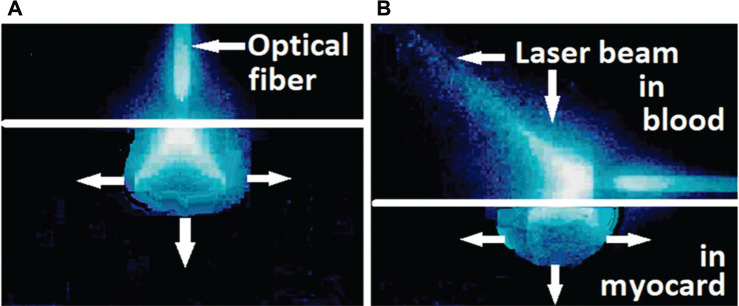
Schematic illustration of 1064-nm laser photon scattering in myocardium. **A:** Vertical catheter position with the catheter end-hole in intimate contact with the endocardial surface. The conically shaped fiber tip is protected in the catheter head 1.0–1.2 mm from the head end-hole. **B:** Horizontal catheter position: a part of the laser beam is dissipated in the bloodstream. Photons that hit the endocardial surface are scattered into the myocardium. Horizontal line, endocardial surface.

**Figure 10:**
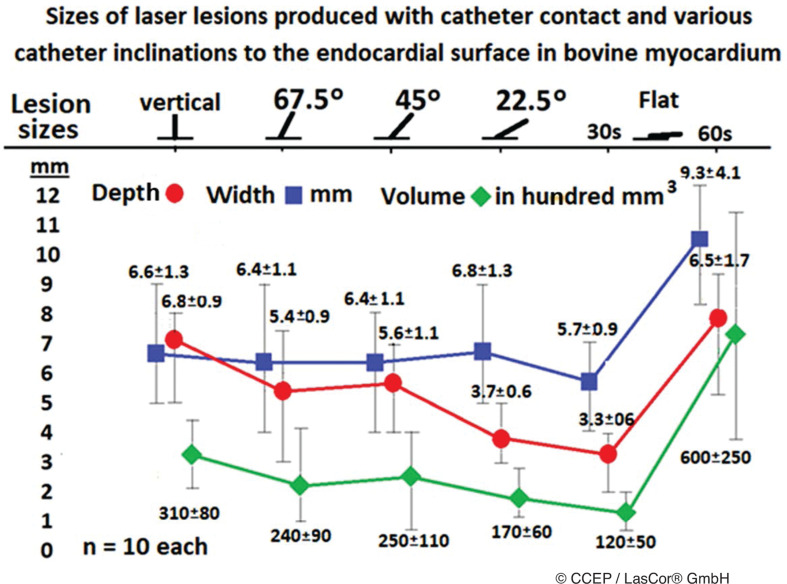
Influence of catheter orientation on laser lesion formation. Mean values of lesion sizes produced at various catheter inclinations of the RytmoLas^®^ during 30 s of radiation time, extended to 60 s during radiation with the catheter in the flat position.

Catheter orientation toward the endocardial area may influence lesion formation. By using the RytmoLas^®^, deep, clear-cut lesions and contiguous stripes of myocardial coagulation can be achieved within seconds, regardless of catheter orientation on the endocardial area.^22^ Laser photons with a 1064-nm wavelength are scattered diffusely in the myocardium regardless of the laser beam angle impact on the endocardial surface **(Figure 9)**. Catheter orientation on the endocardial surface is not a major determinant of lesion size or quality. Even in a flat catheter position, large transmural lesions can be achieved by lengthening radiation times **(Figure 10)**.

#### Energy settings and dosimetry

**Figure 11:**
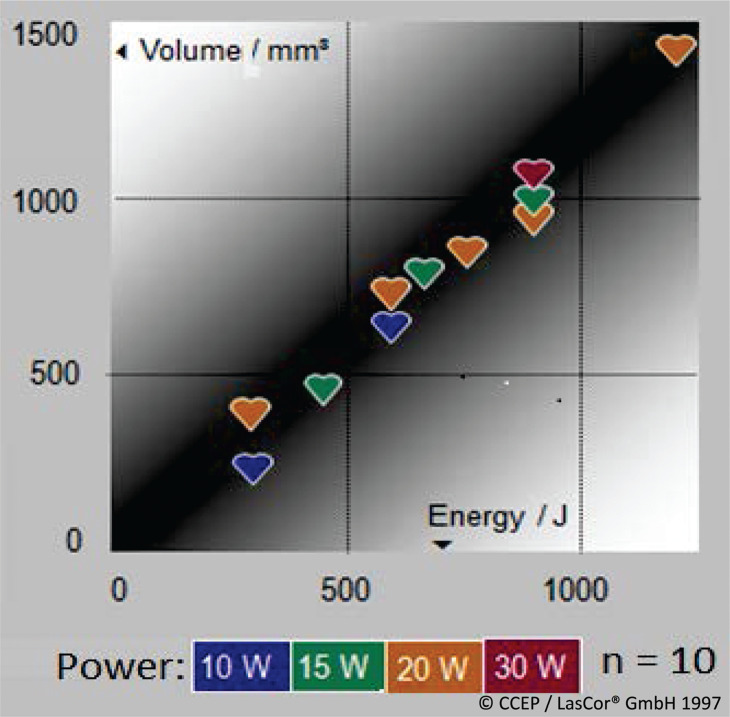
Influence of energy application on laser lesion formation. Linear increase of lesion with the increase of 1064-nm laser energy applied in the beating heart of dogs by using RytmoLas^®^.

**Figure 12:**
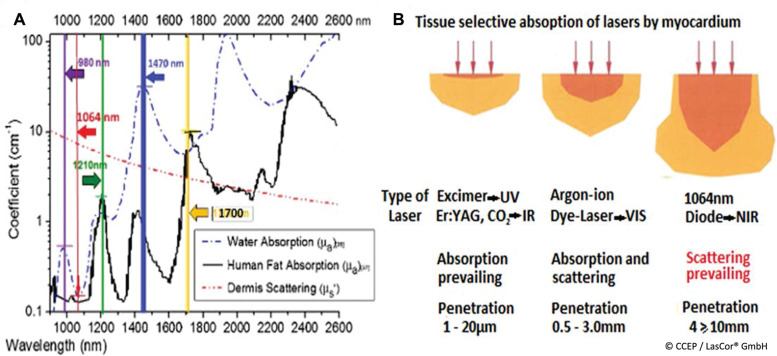
Absorption coefficient in water and tissue-selective absorption of 1064 nm. Comparisons of **(A)** absorption coefficient in water and **(B)** of lesion sizes of various laser wavelengths.

The sizes of laser lesions depend on the energy delivered to the myocardium. Dosimetry shows an approximately linear relationship between laser energy and lesion volumes. The best results have been achieved by using the continuous wave 1064-nm laser wavelength **(Figure 11)**. With its low absorption in water, the main component of myocardium, and moderate absorption in blood as compared to the other laser wavelength **(Figure 12A)**, and with its tissue-selective absorption in myocardium, the 1064-nm wavelength in the near-infrared spectral range is particularly suitable for extensive lesion formation in myocardium **(Figure 12B)**.

**Figure 13:**
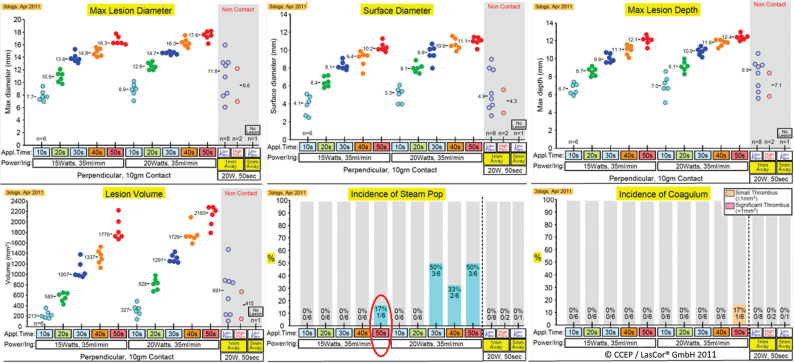
In vivo laser application in a thigh muscle model in anesthetized dogs. Sizes and volumes of lesions, and incidences of steam pop and coagulum after various laser energy settings applied.

By applying various energy settings ranging from 15 W/10 s to 20 W/50 s in an in vivo tight muscle model, the upper limit for the occurrence of steam pop has been found to be 15 W/50 s **(Figure 13)**. Below this “safety level,” when applying energy settings limited to 15 W/40 s, the mean values achieved after six laser impacts were as follows: lesion diameter of 14.8 mm, surface diameter of 10.2 mm, depth of 12.1 mm, and a volume of 1337 mm^3^ without pop. In none of the laser impacts did a coagulum occur, and this confirms that the laser method is not thrombogenic. Lesions were also produced with the catheter tip at a distance from the irradiated spot without thrombus formation. Here, the large variation of lesion sizes can be explained by the circulating pulsatile bloodstream in the experimental setting with dissipation of laser energy into the blood.

### Some anatomic considerations

#### Protection of coronaries

**Figure 14:**
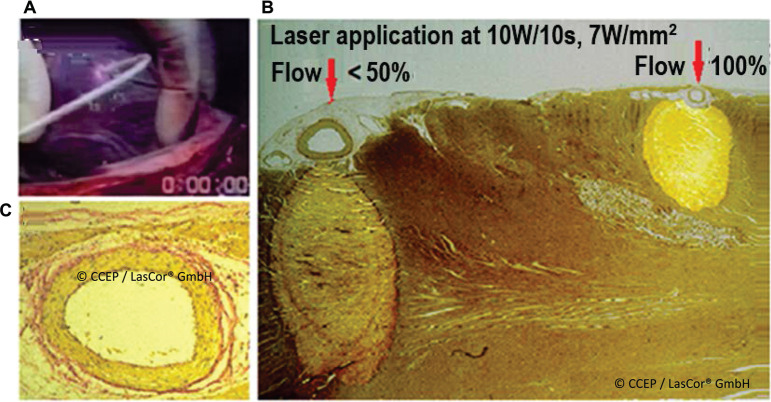
Laser catheter application aimed at epicardial coronaries. **A:** Video image during epicardial laser catheter application aimed at the right coronary artery on a beating dog heart. **B:** Laser lesion achieved during the right coronary flow of <50% and during a normal flow aimed at the circumflex coronary. Arrows indicate catheter orientation. **C:** Slight inflammatory infiltration of the adventitia of the irradiated circumflex artery.

Laser applications do not affect coronary blood flow. Only the small coronary arteries contained within the laser lesion are coagulated without any effect on the myocardial blood supply outside the lesion.^23^ Laser application aimed at the epicardial coronary produces a myocardial lesion through the vessel without damaging the vessel itself because the vessel walls do not absorb photons of the 1064-nm laser light. However, coronary blood flow cools the vessel, thereby significantly reducing the volume of myocardial lesions. The vessel intima and media remain undamaged; only the adventitia shows a slight inflammatory infiltration **(Figure 14)**.

#### Scarred myocardium

**Figure 15:**
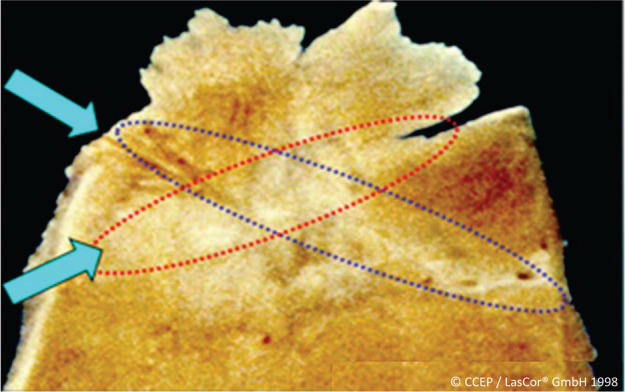
Experimental laser ablation of scarred ventricular myocardium. Ventricular laser scars crossing through each other in a dog heart produced at intervals of 3 months. Arrows indicate the assumed direction of the laser beam during laser application.

In patients with ventricular tachycardia (VT) and ischemic cardiomyopathy and after myocardial infarction, ventricular scars may protect remnant arrhythmogenic myocardium contained in the scars. Laser-induced scars crossing through each other, produced in a dog model, showed penetration of laser photons through scarred myocardium **(Figure 15)**. Laser applications at 10–20 W/15–60 s produced transmural ventricular lesions in scarred ventricular myocardium, suggesting an effective laser treatment of VT in patients with normal and ischemic heart disease.^24^ Ablation for VT is superior to any anti-arrhythmic drug we have available. However, a disappointing finding is that many patients had a recurrent VT; therefore, the focus is on achieving better outcomes.^25^ In this regard, the laser photons scattering through scars and the selective absorption of photons in remnant myocardium encased in the scar can transform fringe and lacunar scars into homogenous solid volumes of lesions devoid of electrical activity and a negligible risk of further sustaining reentry pathways or arrhythmogenic foci within the treatment area.

## The RytmoLas^®^ technology

### Laser catheters

**Figure 16:**
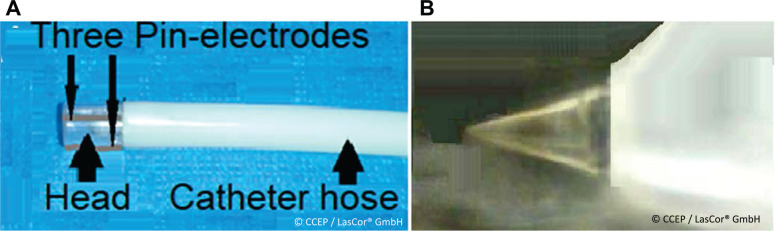
RytmoLas^®^ catheter distal end and optical fiber tip. **A**: RytmoLas^®^ distal end showing its transparent catheter head with the three cable electrodes. **B:** The conically shaped optical fiber tip mounted coaxially and protected inside the catheter head at 1.0–1.2 mm from the catheter end-hole.

The single laser ablation catheter, RytmoLas^®^, is an 8-French flexible plastic tube with a total length of 3.0 m and a working length of 115 cm that is designed for non-contact, open-irrigated, electrode laser, ablation procedures guided by HD laser-mapping in the cardiovascular system for the treatment of cardiovascular diseases. It is a spot-by-spot ablation device capable of creating transmural atrial or ventricular lesions or circumferential ablation of a 5.0–10.0-mm vessel segment. This is made possible by the “donut”-like circular radiation spot produced by the divergent beam emanating from the canonically shaped optical fiber tip protected within the catheter head **(Figure 16)**. The catheter is also designed with three tip electrodes orientated longitudinally on the catheter head at 2.0-mm interelectrode distances, which allow for HD mapping during intracardiac catheter exploration for diagnostic and ablation procedures—that is, HD laser-mapping.

**Figure 17:**
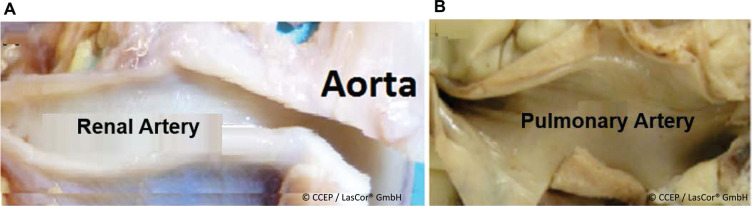
Open arteries after four contiguous continuous-wave 1064-nm laser applications. No lesions are visible on **(A)** the intima of the right renal artery in a pig and **(B)** in the left and right pulmonary artery branches in a sheep produced by using the laser catheter RytmoLas^®^. Four applications in each animal were performed. Anteroposterior view.

RytmoLas^®^ was designed for the ablation of arrhythmogenic substrates. However, perivascular renal and pulmonary artery nerves were also found to be specifically sensitive to the photons of 1064-nm laser light.^26,27^ Notably, perivascular nerves can be coagulated without damage to the vessel layers **(Figure 17)**. For these “all-in-one” laser applications of the RytmoLas^®^, separate instructions for use (IFU) are available:

1. For cardiac arrhythmia and cardiomyopathies (hypertrophic obstructive cardiomyopathy and Chagas cardiomyopathy) ablation: see the IFU for RytmoLas^®^.
2. For resistant systemic and pulmonary hypertension: see the IFU for HypertenoLas^®^ (LasCor GmbH).
3. For varicose vein ablation: see the IFU for ScleroLas (LasCor GmbH).

For vascular access and catheter manipulation in the cardiovascular system, commercially available long steerable sheaths and introducer sets, eg, AGILIS (Abbott, Chicago, IL, USA), can be used after puncture of a vessel (Seldinger technique) in the groin or an arm.

**Figure 18:**
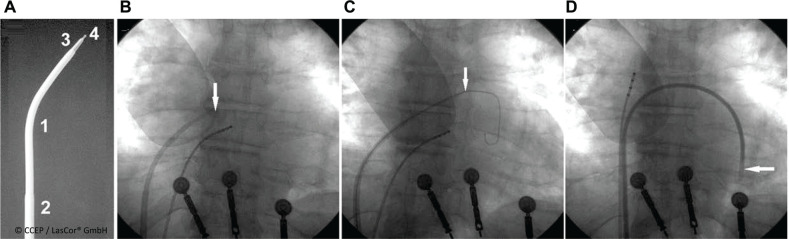
Side-selective atrial transseptal laser puncture procedure. **A:** Steerable sheath (1, 2), dilator tapered tip (3), and optical fiber tip (4) extending 2.0 mm from the dilator end-hole. **B:** The dilator tip points toward the area of interest, where the side-selective transseptal puncture is aimed at (arrow). **C:** The dilator tip (arrow) after septal puncture is advanced into the left atrium and the guidewire is deployed in the left atrium. **D:** The sheath is advanced into the left ventricle (arrow). The dilator and the guidewire are removed.

For the side-selective transseptal laser puncture procedure **(Figure 18)**, an optical fiber set, ISPunctureLas^®^ (LasCor GmbH) may be used, connected to the laser generator CardioVascLas^®^ (LasCor GmbH) and by choosing the assistant *Transseptal Puncture* preset in the laser software. Laser puncture may be performed without complication and with a first-attempt success,^28^ as described in the IFU for ISPunctureLas^®^.

**Figure 19:**
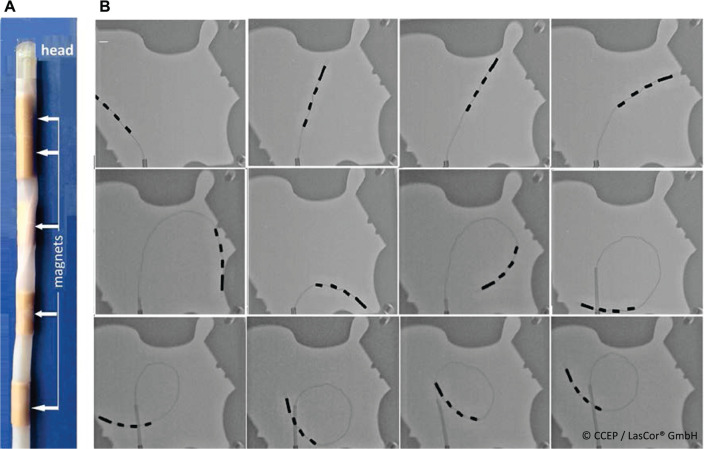
Magnetic navigation of the RytmoLas^®^m in the Stereotaxis system. **A:** The distal end of the laser catheter RytmoLas^®^. with its magnets in the transparent silicon tube. **B:** Various positions and configurations of the catheter’s distal end during magnetic navigation.

Besides manipulation with a steerable sheath, robotic navigation of the RytmoLas^®^ by using the mechanical robotic system of Hansen Medical (Mountain View, CA, USA) has also been tested. More recently, magnetic navigation in the Niobe system (Stereotaxis, St. Louis, MO, USA) was tested successfully by using a catheter variant provided with a flexible tube with magnets, the RytmoLas^®^ **(Figure 19)**. The application is described in the IFU.

### Laser generators

Initially, a 1064-nm solid-state continuous-wave Nd:YAG laser generator, the CardioVascLas^®^, was manufactured and tested for laser ablation. The laser weight was about 110 kg and water-cooled, which, after several years, was replaced by a 10-kg air-cooled Dornier Medilas (Dornier MedTech GmbH, Wessling, Germany) diode laser.

**Figure 20:**
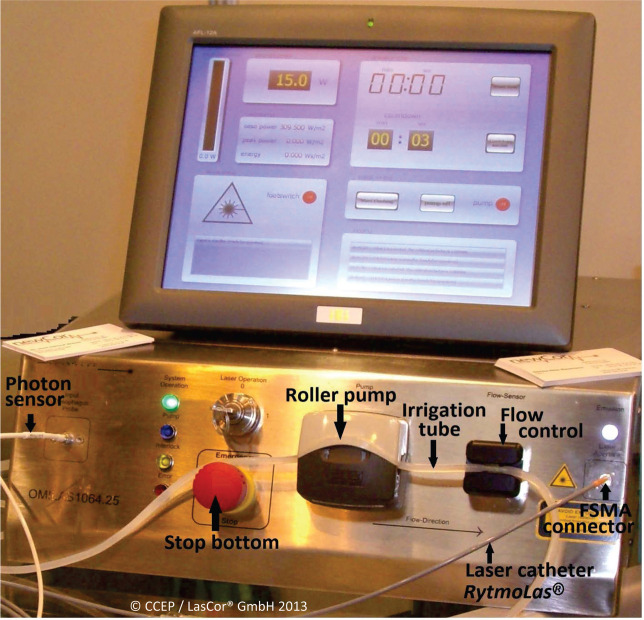
CardioVascLas^®^ compact diode laser with an open screen.

After production of this laser was stopped by Singapore Technologies (Singapore), it was replaced by a novel diode laser provided with multiple functions **(Figure 20)**. Eventually, after further optimizing laser functions with new software, a compact 8-kg diode laser, the IriFlowLas^®^ (LasCor GmbH), was manufactured.

All the lasers were provided with a roller pump for catheter irrigation that was automatically controlled via the laser footswitch. After replacing the air with saline flushing of the catheter at a rate of 80 mL/min, the irrigation is switched to a 10 mL/min continuous “low flow.” The catheter is then introduced and advanced in the steerable guide already positioned with its distal end in the right atrium (RA). The 10 mL/min “low flow” increases automatically to 35 mL/min “high flow” via the laser foot switch during laser application, creating a clear pathway for the laser beam to the target area.

Via the touchscreen of the laser, three assistant systems are available. One screen is for the side-selective interatrial transseptal puncture with 5.0 W/5.0 s, without irrigation flow. The second is for the laser ablation of the thin-walled myocardium (≤4.0 mm thickness), such as for atrial myocardium or for atrial fibrillation (AF) ablation with 10 W/10 s. The third is for ablation of the thick-walled myocardium (≤4 mm thickness), such as for ventricular arrhythmias with 15 W/15 s. In both the latter cases, the 10 mL/min continuous “low flow” is automatically increased to a 35 mL/min “high-flow” saline irrigation during laser application.

After the preset times of 10 or 15 s of laser application, respectively, the laser stops automatically, but radiation can be repeated by pressing the foot switch again, and “low flow” will again switch automatically to “high flow.” There is no combination of the laser generator CardioVascLas^®^ with another power source or another irrigation pump.

### Roller pump

The IriFlowLas^®^, an accessory of the CardioVascLas^®^, is a 6.7 kg, 285- × 262- × 155-mm irrigation pump designed for the irrigation of open-irrigated laser catheters. Catheters are flushed with heparinized saline at room temperature at a rate of 80 mL/min for air removal prior to insertion into the patient. The flow rate is then set on the touchscreen of the pump at a 10-mL/min continuous “low flow” and to a 35-mL/min “high flow” during laser application. For the use of open-irrigated catheters in the blood-filled cardiovascular system, continuous catheter irrigation is a must.

## Clinical data

### Performance

**Figure 21:**
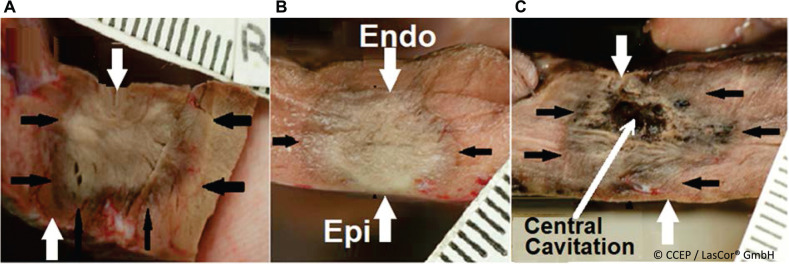
**A:** Lesion was achieved at 20 W/20 s; lesion was non-transmural (partial) because electrical potential amplitudes in the high-density laser-mapping electrogram were reduced approximately 70% but not abolished. **B:** Transmural lesion was achieved after permanent abolishment of electrical potential amplitudes in the high-density laser-mapping electrogram in 26 s, but laser application was extended 4–30 s to assure permanent abolishment of electrical potentials and to achieve a clear-cut homogenous transmural lesion limited to the myocardial wall. **C:** Extending laser application 10 s after permanent abolishment of potential amplitudes in the high-density laser-mapping will result in pop with intramural cavitation. Lesions are indicated by black arrows.

HD laser-mapping is usable because the emitted photons of 1064-nm laser light do not interfere with electrophysiologically mapping principles. The endocardial electrical potentials are visible in the HD laser-mapping electrogram without electrical hum during laser application. Their gradual abatement visualizes lesion formation in the myocardial wall on the monitor and allows for immediate and real-time verification of the success of treatments. With the permanent abolishment of electrical potentials, a transmural lesion is achieved. The correlation coefficient is 0.9.^29^ The timely termination of laser application will stop further growth of the lesion that will remain limited to the myocardial wall, avoiding collateral damage to the adjacent anatomical structures, including the esophagus, lungs, and nerves. Extending laser application beyond this limit will result in deleterious effects on the heart **(Figure 21)**. However, of importance is the observation that an intramural pop never resulted in rupture of the ventricular wall; endo- and epicardial lesion layers were always contiguous.

### Reversible laser effects

**Figure 22:**
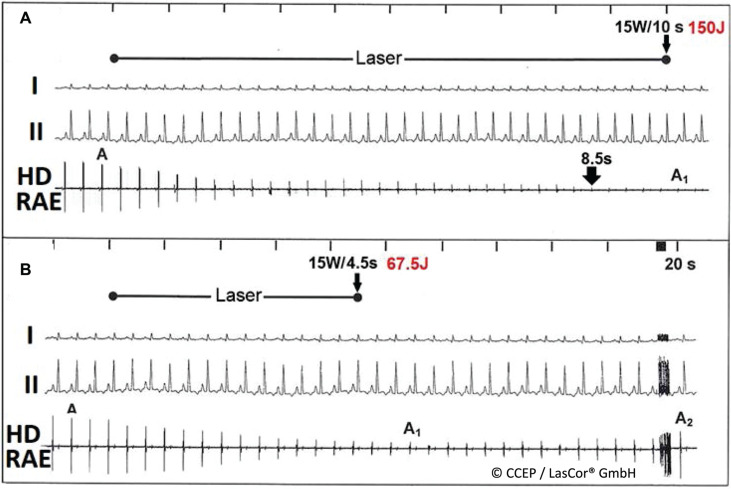
High-density laser-mapping during right atrial wall laser application. **A:** Permanent abolishment of electrical potential amplitudes in the high-density laser-mapping after 8.5 s. **B:** Recovery of potential amplitudes from A_1_ to A_2_ to baseline with laser stop after 4.5 s. *Abbreviation:* HD RAE, high-density right atrial electrogram (HD laser-mapping).

**Figure 23:**
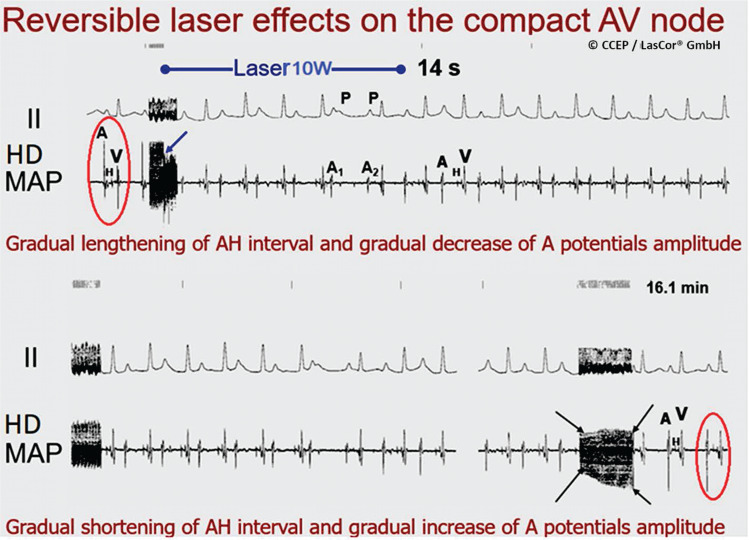
Oblique arrow, top: dwindling of potential amplitudes with the start of laser application. Oblique arrows, bottom: gradual increase of the electrical potential amplitudes. Note the normalization of conduction after 16 min. *Abbreviations:* II, surface lead electrocardiogram; A, atrial; V, ventricular, H, His-bundle potentials; HD MAP, high-density laser-mapping.

**Figure 24:**
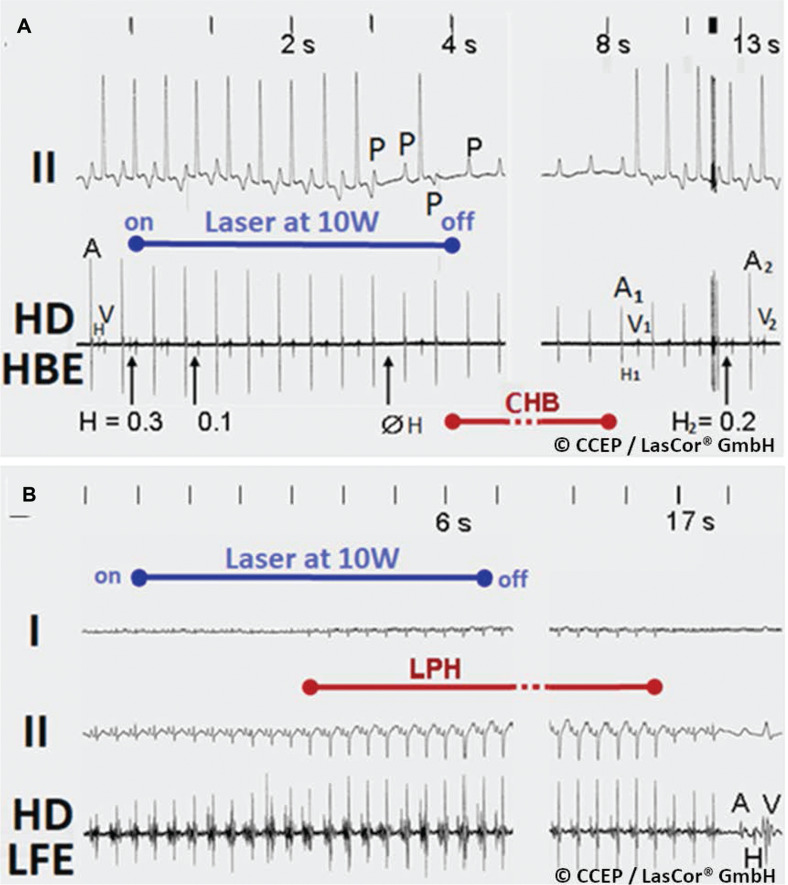
Reversible laser effects on the His-bundle and the left fascicle. High-density laser-mapping of the His-bundle and left fascicle. **A:** Laser application at 10 W/4 s aimed at the atrial segment of the His-bundle abolished the potential, inducing complete His-block (ØH). Timely stop of radiation after 4 s allows for recovery of conduction and of the His-potential amplitudes to baseline (H_2_). **B:** Laser application aimed at the left posterior fascicle, producing conduction delay through the bundle with axis deviation. Stopping of radiation after 7 s allows for normal conduction after 10 s. *Abbreviations:* I, II, surface lead electrograms; A, atrial; H, His-potentials; LPH, left posterior hemiblock; HD HBE, high-density His-bundle; p, P-wave; V, ventricular.

**Figure 25:**
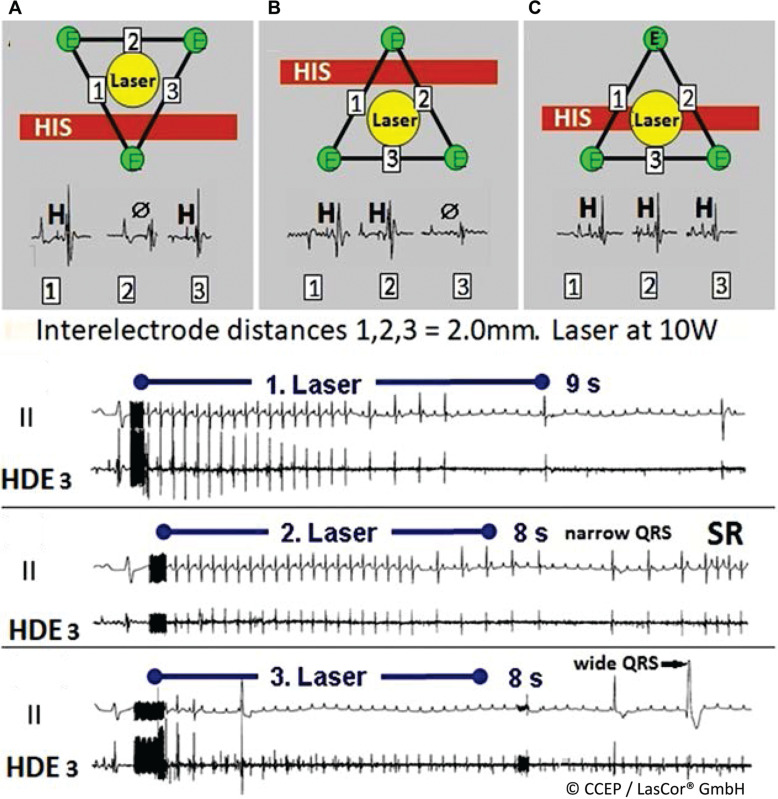
High-density laser-mapping during His-bundle laser application. Schematic Illustration of catheter positions. **A, B:** During laser application aimed at the His-bundle with the catheter more laterally with bipolar His recordings (H) from 1, 3 and 1, 2 positions that are overriding the His. **C:** Catheter position with the electrodes (E) close to the His with H-potential recordings in all three bipolar electrograms 1, 2, and 3. **A:** Gradual abatement of ventricular potentials close to the penetrating bundle. His-block in 5 s with recovery of conduction (after 2 min). **B**: His-block after 6 s with recovery after 7 s, narrow QRS, and sinus rhythm. **C**: Permanent His-block in 2–3 s of radiation; wide QRS complexes. *Abbreviations:* Ø, no His-potential; II, surface lead electrocardiogram; HDE, high-density electrogram; SR, sinus rhythm.

In contrast to the permanent abolishment of electrical potential amplitudes after the premature stop of radiation, amplitudes recover gradually up to their baseline amplitudes **(Figure 22B)**. In cases of complete recovery of electrical potential amplitudes in atrial HD laser-mapping, there was no anatomic correlation found in the irradiated atrial area. It can be assumed that initially induced slight edema and intramural hemorrhage are completely reversible. This is of crucial importance for the protection of the normal conduction system **(Figures 23 and 24)** and helps prevent permanent heart block.^30^ Electrical recordings from a very small endocardial area **(Figure 25)** allow for accurate visual evaluation of laser effects on the irradiated substrate on the monitor and for distinction between the normal conduction system and the arrhythmogenic substrates that help save normal structures of the heart. The unique benefit is that HD laser-mapping allows for a systematic approach with simultaneous validation of initial success during laser application and for immediate and real-time verification of the success of treatment.

### Safety

After the first successful HD mapping–guided direct current shock ablation of an arrhythmogenic substrate in August 1982,^31^ various power sources were tested. Eventually, after numerous laboratory and in vitro tests, along with in vivo animal experiments, the 1064-nm laser light was found to be most promising for safe and effective CVLA, including of arrhythmias.

After a phase I clinical study in four patients (1 with an accessory pathway in 1988 and 3 with AV nodal re-entry and atrial flutter),^32,33^ a phase II clinical study was performed in 1997 in 10 patients with AV nodal re-entry tachycardia.^34^ Subsequently, 69 patients with various cardiac arrhythmias were treated with the laser method in a phase III multicenter clinical study trial.^35^

In addition, laser catheter ablation of long-lasting persistent AF in patients with various comorbidities^36^ and laser catheter modulation of the sinus node in the cure of inappropriate sinus tachycardia were performed.^37^ In all the clinical tests, excellent procedural safety was achieved without any adverse events in patients and during in vivo animal experiments. A major contribution to the favorable safety profile of the laser treatment can be assumed by the non-contact mode of energy application, without CF but with visual control of laser effects on the monitor during laser application—that is, the HD laser-mapping. Outstanding advantages were the tissue specificity of the 1064-nm laser; that the laser is not thrombogenic; and that the laser does not endanger the coronaries, kidneys, esophagus, lungs, or vagus nerve. On the contrary, it modulates retrocardiac ganglion plexi, improving the results of AF ablation. The follow-up was ≥5 years up to lifelong.

Easy handling, like the routine ablation techniques used worldwide, with a very short learning curve, is also an important safety aspect. No complications occurred during the more than 1000 laser impacts performed in in vivo animal experimental studies in over 100 animals, mainly beagles, except in one pig during renal sympathetic denervation due to inappropriate handling of catheter irrigation.

### Efficacy

Photons of 1064-nm wavelength are highly tissue-selective, representing a very powerful energy source. Without control, laser application may result in deleterious effects.^38^ For ablation of arrhythmias in the thick-walled myocardium, eg, VTs, the energy level must be set below the upper safety limits of 15 W/50 s/35 mL/min (**Figure 11**, incidence of steam pop). HD laser-mapping with visual control of immediate laser effects is therefore a prerequisite for the use of the method.

**Figure 26:**
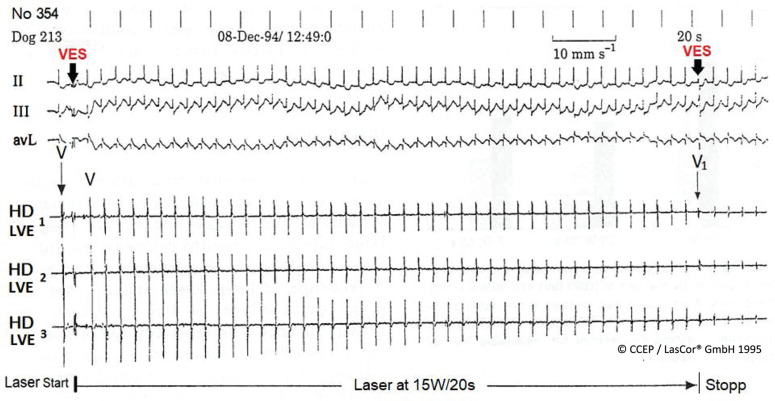
High-density laser-mapping during LV 1064-nm laser catheter application. Gradual abatement of ventricular potential amplitude VV_1_ recorded via the three tip electrodes of the RytmoLas^®^ without electrical hum during laser application. Start and stop of the laser are marked by “VES.” Transmural lesion (see **Figure 3**). *Abbreviations:* II, III, aVL, surface lead electrocardiograms; HD, high-density; LV, left ventricle; VES, ventricular extrasystole.

**Figure 27:**
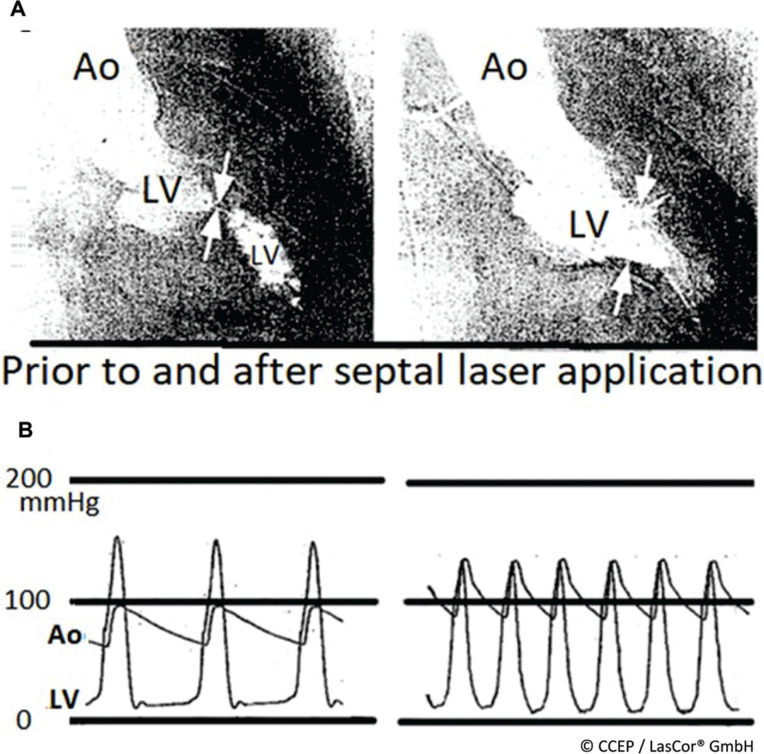
Laser ablation of left ventricular outflow tract obstruction hypertrophic obstructive cardiomyopathy. **A:** Left ventricular right anterior oblique angiogram in a patient with severe subaortic obstruction prior to (left) and after laser ablation at 15 W/25 s (right) aimed at the right side of the interventricular septum showing the maximum obstruction (kissing arrows). **B:** Femoral artery and left ventricular pressure curves in mmHg prior to (left) and after (right) laser ablation of the left ventricular outflow tract obstruction.

If permanent abolishment of potential amplitudes is not achieved in the preset 15 s when the laser stops automatically, a subsequent laser impact is to be delivered by pressing the foot pedal again. However, it must be released immediately when the abolishment of potentials is achieved **(Figure 26)**. Transmural ventricular lesions can ablate hypertrophic obstructive myocardium by releasing subaortic outflow tract obstruction. Laser application aimed at the interventricular septum from the right ventricular side can avoid risky retrograde arterial catheterization **(Figure 27)**. However, for laser ablation of Chagas VT, retrograde left ventricular catheterization or transseptal puncture may be needed. *Trypanosoma cruzi*, the pathogen of Chagas VT, is sensitive to 1064-nm photons such that the laser could be an intriguing alternative to surgery or septal alcohol ablation.^39^

HD laser-mapping avoids lesion extension in adjacent healthy tissue. Transmural ventricular lesions limited to the myocardial wall can be achieved within seconds; the laser is a low-power, short-duration ablation method. VT ablation can be performed successfully in trabeculated and scarred myocardium as well while saving at the same time the coronary blood flow outside the compact, clear-cut lesion. Patients with ischemic cardiomyopathy usually have a discrete scar. This presentation differs from many non-ischemic cardiomyopathies, which may have multiple scars or scars that do not come from a previous myocardial infarction. The technical challenges and complexities of ablation are much higher than they are for ischemic cardiomyopathies.

**Figure 28:**
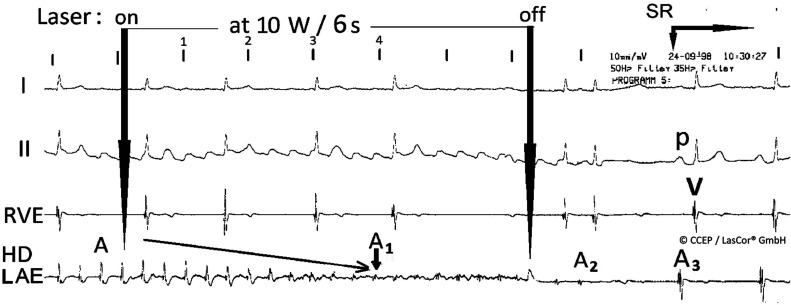
High-density left atrial laser-mapping during ablation of chronic persistent atrial fibrillation. Gradual abatement of atrial potential amplitudes (AA1) without electrical hum with abolishment in 4 seconds, denoting a transmural left atrial posterior wall lesion. Transition to sinus rhythm (A2A3). *Abbreviation:* HD LAE, high-density left atrial (laser-mapping).

**Figure 29:**
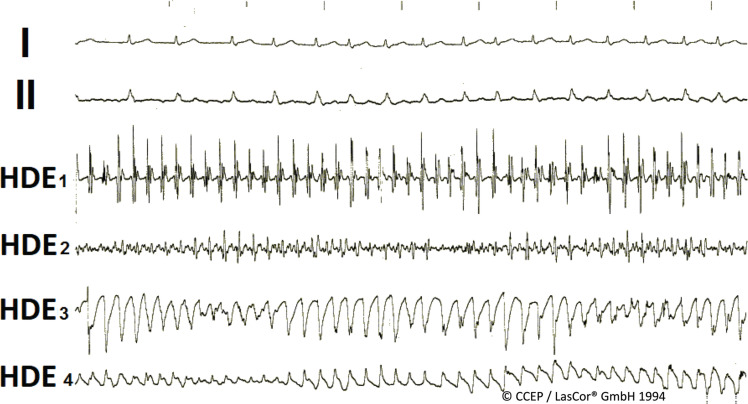
High-density mapping during right and left atrial catheter exploration. High-density mapping electrograms recorded from various endocardial areas of the atrial walls (HDE_1–4_) during catheter exploration in a patient with long-lasting chronic persistent atrial fibrillation. Start of ablation in HDE_1_. *Abbreviation:* I and II, surface lead electrocardiograms.

For laser applications in thin-walled regions, eg, atrial myocardium, ablation parameters are preset at lower values (eg, 10 W/10 s/35 mL). If abolishment of potential amplitudes is achieved earlier, the foot pedal must be released before 10 s **(Figure 28)**. During catheter exploration of the atria in patients with AF, endocardial electrograms differ **(Figure 29)**. The best effects of laser ablation may be achieved by starting ablation around the highest regular potentials (**Figure 29**, HDE_1_). Largest-amplitude ablation is also the optimal approach for ablation of atrial flutter.^40^ Laser application aimed at areas with smaller complexes or torsade-like electrograms is less effective, increasing the number of ablation attempts and lengthening the procedure time.

**Figure 30:**
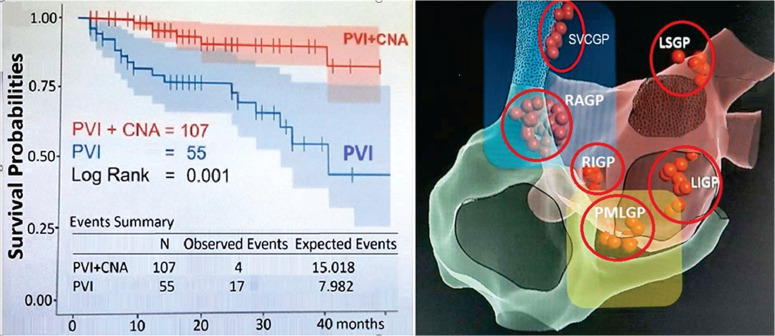
A: Impact of cardioneuroablation on atrial fibrillation ablation. Figure developed using data from Pachon-M JC, Pachon-M EI, Pachon CTC, et al. Long-term evaluation of the vagal denervation by cardioneuroablation using Holter and heart rate variability. *Circ Arrhythm Electrophysiol*. 2020;13(12):e008703. **B:** Value of ganglionated plexi ablation in atrial fibrillation ablation. *Abbreviations*: GP, ganglion plexi; location of cardiac GP: LS, left superior; LI, left inferior; PML, paraseptal-medial-left; RA, right anterior; RI, right inferior.

**Figure 31:**
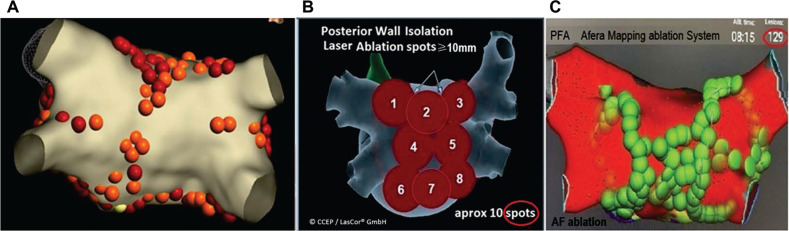
Comparison of radiofrequency, laser, and pulsed field ablation lesions. A schematic representation of each is shown. **A:** A circumferential pulmonary vein isolation and pulmonary wall box lesion set created using radiofrequency energy; note the smaller lesion size relative to those created with laser ablation or pulsed field ablation. *Reprinted with permission from*: Dubey A, Amhed A, Patel H, et al. Epicardial potential in the left atrium during posterior wall isolation in persistent atrial fibrillation. J Innov Cardiac Rhythm Manage. 2023;14(7):5510–5513. **B:** Spot sizes of left atrial posterior wall laser lesions are typically larger, requiring significantly fewer lesions (~10). **C:** Affera™ mapping–guided pulsed field ablation lesions (n = 129) for atrial fibrillation ablation. *Source*: Medtronic.

Our high AF ablation success rate of >90%, with follow-up of >5 years, can be explained by our systematic approach, starting with ablation of the left atrial (LA) posterior wall, a general approach, followed by RA and septal areas,^34^ all areas of cardiac ganglion plexi locations. The laser may cause modulation of ganglion plexi, as shown by the combination of pulmonary vein isolation (PVI) and cardioneuroablation **(Figure 30)**.^41^ The laser ablation of 1064-nm wavelength at 15 W for <30 s aimed at the posterior LA wall does not damage the esophagus, but laser effects on the ganglion plexi reduce AF inducibility.^42^ Laser ablation of ganglion plexi zones is feasible and reduces the inducibility of AF. No change in the atrial effective refractory period may be detectable following ganglion plexi zone ablation when performed from the RA. PVI is not enough because, without modulation of the ganglion plexi, success rates are substantially lower. AF ablation methods that do not modulate ganglion plexi during AF ablation or are associated with transitory and short-lasting effects on the autonomic nervous system, which recover almost completely within a few minutes after ablation,^43^ will yield limited results of AF ablation, requiring additional ablation to be added to PVI.^44^ Durability of laser lesions is independent of the configurations of atrial myocardial tissue, of bridges, or of trabeculated areas. Extensive contiguous transmural overlapping laser lesions will result in gapless PVI **(Figure 31)**. PVI plus ganglion plexi ablation confers superior clinical results with less ablation-related LA flutter and reduced AF recurrence compared to PVI plus linear lesion ablation.^45^ Biatrial cardioneural ablation provides incremental benefits after both RA and LA ablation. Starting ablation in the LA provides the most significant effect on neural modulation.^46^

## Future clinical implications

The CardioVascLas^®^ system, which was initially designed for laser catheter ablation of cardiac arrhythmias, represents an innovative approach in the field of minimally invasive cardiovascular interventions aimed at treating cardiac arrhythmias, Chagas disease (the sensitive parasite *Trypanosoma cruzi*), cardiomyopathies, and resistant systemic and pulmonary hypertension. CVLA is performed under normothermic conditions. Lesions are created by tissue-selective absorption of the 1064-nm photons in the targeted tissue, whereas the catheter itself is not heated up and does not transmit heat. Treatment is performed under normothermic conditions while avoiding interference with electrophysiologic principles. Immediate and real-time verification of the success of treatment is extremely beneficial. The 1064-nm laser wavelength is the only true tissue-specific power source. It is not thrombogenic; does not produce microemboli, stroke, or hemolysis with potential kidney injury, irreversible conduction block, or coronary spasm; and there is no tissue vaporization with crater formation in the irradiated myocardial or vessel walls.

The laser catheter RytmoLas^®^ used for the treatment of all the aforementioned indications is an open-irrigated electrode laser-mapping and ablation catheter—all-in-one—with non-contact laser energy transmission to the target through a clear pathway of saline flow without CF on the irradiated area. After HD mapping and localization of the culprit arrhythmogenic area, ablation is performed by laser application with the same catheter, and laser effects are visualized on the monitor by gradual abatement of local potential amplitudes during laser application. This unique approach—HD laser-mapping—allows for limitation of lesion formation on the target tissue, sparing all adjacent tissues, including the esophagus, lungs, and vagus. This is a very important characteristic, a crucial safety aspect of the laser method.

Its excellent safety profile and unrivaled high success rate substantially simplify the procedure, allowing for the waiver of additional sophisticated and expensive mapping systems, for shortening of the procedure by reducing radiation times and minimizing redo interventions. It is the shortest overall procedure duration. Catheter mapping was performed under X-ray control except in one center, where the EnSite NavX system (Abbott, Chicago, IL, USA) was successfully used, which is an option. With the request for design to cost for competitive products, the multipurpose, unsophisticated CardioVascLas-System^®^ (LasCor GmbH) is a highly competitive cardiovascular treatment option.

The reviewer of the Notified Body classified the laser method as a superior technique compared to other ablation systems. Advantages of the laser method were confirmed by the editor-in-chief of the *Journal of Innovations in Cardiac Rhythm Management*^47^ and by experts of the Mayo Clinic.^48^ Electrophysiologist J. Borbola stated: “[a] laser ablation system should be available in electrophysiological laboratories. This technique will have wider applications soon.”^49^

Laser ablation guided by HD laser-mapping represents disruptive technology in the field of cardiac electrophysiology, with the potential to become an all-pervasive procedure and a paradigm shift in the treatment of cardiovascular diseases, including cardiac arrhythmias, cardiomyopathies, and resistant systemic and pulmonary hypertension. The accumulating body of evidence supports its efficacy and safety, positioning the laser, a key technology, as more than just a viable alternative to traditional ablation techniques.

The system and components are patented (EU, USA, Russia/Commonwealth of Independent States) and have UDI-DI identification, trademark numbers, and EO-sterilization validation. The laser method has a large potential for further development, eg, artificial intelligence (AI). Especially, HD laser-mapping, with its gradual abatement and eventually the abolishment of electrical potential amplitudes, may represent an approach for AI for controlling laser treatment automatically. Using AI could possibly substantially simplify and shorten the procedure. However, clinical integration of AI in AF management is still an evolving field.^50^

### Limitations

After the expiration of the catheter certificates in May 2023, updated technical documentation for a new MDR approval was submitted and accepted by TÜV SÜD in Munich. The production line was ended, and work concentrated on the development of a new compact 1064-nm diode laser system, the new CardioVascLas^®^, which was successfully implemented in July 2024.

## Conclusions

Unprecedented safety and efficacy position the CardioVascLas^®^ system as a superior alternative to all other ablation techniques. It is the only true tissue-specific ablation technique that is not thrombogenic. It is performed under normothermic conditions, producing lesions within seconds under visual control. However, more research and clinical experience, including randomized controlled trials, are still pending.
